# Long-term effects of SARS-CoV-2 infection and vaccination in a population-based pediatric cohort

**DOI:** 10.1038/s41598-024-84140-6

**Published:** 2025-01-23

**Authors:** Jakob Höppner, Christoph Maier, Anne Schlegtendal, Anna Hoffmann, Astrid Petersmann, Thomas Lücke, Nicole Toepfner, Folke Brinkmann

**Affiliations:** 1 University Children’s Hospital, St. Josef-Hospital, Ruhr-University Bochum, Bochum, Germany; 2 Institute for Clinical Chemistry and Laboratory Medicine, University Medicine Oldenburg, Oldenburg, Germany; 3 Institute for Clinical Chemistry and Laboratory Medicine, University Medicine Greifswald, Greifswald, Germany; 4 Department of Pediatrics, Faculty of Medicine and University Hospital Carl Gustav Carus, Technische Universität Dresden, Dresden, Germany; 5 Section of Pediatric Pneumology, Department of Pediatrics, University of Lübeck, Campus Lübeck, Ratzeburger Allee 160, 23538 Lübeck, Germany; 6 Airway Research Center North (ARCN), Germany, Member of the German Center for Lung Research (DZL), Lübeck, Germany

**Keywords:** SARS-CoV-2, Children, Seroprevalence, Follow-up, Vaccination, Diseases, Health care

## Abstract

During the omicron wave of the COVID-19 pandemic and with SARS-CoV-2 vaccines becoming available, seroprevalence rates rose in children and adolescents. This study investigated the impact of both SARS-CoV-2 infections and vaccinations on the incidence of acute and prolonged symptoms in real-world conditions during the transition from the pandemic to the endemic phase. Participants from a pediatric population based seroprevalence study (CorKID study) were followed up at least two and for almost four years by survey of health status features and symptoms suggestive of post-COVID syndrome (PCS). In a subgroup (n = 259) SARS-CoV-2 antibody serology was further investigated. 789 participants of the original CorKID study cohort (n = 2.121; 37.2%) were included. 67.9% reported at least one SARS-CoV2 infection. 46.6% had received one or more SARS-CoV-2 vaccinations. In the vast majority of serologically tested participants antibodies again SARS-CoV-2 spike (98.9%) or nucleocapsid (93.3%) antigen were detected following infection and/or vaccination. At least 30% experienced one unrecognized SARS-CoV-2 infection. The overall health status was comparable between children, irrespective of SARS-CoV-2 infections and similar to pre-pandemic assessment. However, a subset of young adolescents exhibited a decline in physical performance compared to pre-pandemic conditions. After infection, PCS-like symptoms persisted in 7% of the respondents for more than three months and up to four years. SARS-CoV-2 vaccinated participants (47%) reported 12% less acute flu-like infections other than SARS-CoV-2. Nearly all participants developed SARS-CoV-2 antibodies in this longitudinal study through either vaccination or infection during the Omicron wave. About 7% of participants suffered from PCS symptoms, predominately fatigue and exhaustion. Furthermore, participants who received vaccinations against SARS-CoV-2 reported a lower frequency of acute infections during follow-up.

## Introduction

Early in the COVID-19 pandemic, infection rates as well as infection severity among children were controversially discussed. Previously, we performed a population-based cohort study in 2124 asymptomatic children and their parents (CorKID Study)^1,2^ who were included during scheduled and legally required preventive examination in a metropolitan region in Germany between June 2020 to February 2021. Primary endpoint was the SARS-CoV-2 seroconversion rate during the study period. At that time, only 58 children were seropositive for SARS-CoV-2 and 24 (41%) of those reported symptoms. Subsequently, and particularly with the emergence of the omicron variant at the beginning of 2022, infection rates increased across the population. At about the same time, vaccination was also made available for children and adolescents.

Although most SARS-CoV-2 infections in children were mild, some developed either complications including pediatric inflammatory multisystem syndrome (PIMS) or persisting symptoms after COVID 19^3^. Population based data on pediatric cohorts in Germany is essential to evaluate long- term outcomes after SARS-CoV-2 infection and/or vaccination with this now endemic virus.

Therefore, the objective of the current study (CorKID2.0) was to track the health trajectory of a well-characterized cohort of children and adolescents throughout the SARS-CoV-2 pandemic, specifically examining their health evolution during the mid-pandemic phase. Special emphasis was given on the timing and number of infections as well as COVID-19 vaccination effects on disease course.

## Material and methods

### Study design

Participants of the CorKID study were enrolled into the CorKID2.0 follow up study. The study protocol was approved by the Ruhr-University Bochum Ethics Committee (reference BO-EK-156042020). Written informed consent was obtained from each patient, a parent and/or legal guardian. The study was conducted in accordance with the principles of the Declaration of Helsinki. The CorKID study procedure took place as described previously^1,2^. In brief, asymptomatic children and adolescents from 6 months to 18 years of age (and their parents) who attended outpatient pediatric practices for scheduled mandatory routine prevention examinations (U-Untersuchungen) were invited to participate in the study. None of the participants were vaccinated against SARS-CoV-2 at the time of the study.

For CorKID2.0, participants or their parents were contacted via telephone between September 1st and October 31st, 2022. Trained individuals conducted interviews to gather information on their health status and individual experiences throughout the pandemic. This interview included questions about the timing (day, month and year) and history of COVID-19, SARS-CoV-2 vaccinations, infections other than SARS-CoV-2 as well as evidence of persistent symptoms,. In accordance with the National Institute for Health and Care Excellence (NICE) guidelines that were widely accepted at the time of the study design, symptoms after SARS-CoV-2 infection lasting up to one week after infection were classified as prolonged, symptoms lasting longer 4 weeks and up to three months as long-covid syndrome, and symptoms lasting more than three months (12 weeks) as post-covid syndrome (PCS)^4,5^. To assess persistent symptoms, only cases were considered in which a minimum of 3 months had transpired between the interview and the onset of the SARS-CoV-2 infection. Participants experiencing prolonged COVID-19 symptoms that lasted more than three months at the time of the 2022 interview were recontacted in January 2024; with 19 of 31 individuals successfully reached (4.2 years ± 1.2 after the initial SARS-CoV-2 infection). Further, the current state of health, quality of life, fitness and mental state at the time of the follow-up interview were also queried according to the WHO Quality of Life (WHOQOL-BREF) questionnaire^6^.

In addition, all participants were invited to a clinical examination at the University Children’s Hospital in Bochum for SARS-CoV-2-antibody testing. Of those participants, serum samples were analyzed for SARS-CoV-2 spike (S) and nucleocapsid (N) antibodies (ECLIA Anti-SARS-CoV-2, Roche Diagnostics GmbH, Mannheim, Germany). In brief, antibodies (Ab) against the spike (S) protein (S-Ab) occurring after vaccination and/or disease were evaluated quantitatively (positive at a value ≥ 0.8 binding antibody units [BAU]/mL). Antibodies against the nucleocapsid (N) protein (N-Ab) specific for a previous infection were evaluated qualitatively (positive at assay-specific cut-off index value ≥ 1.0).

### Statistical analysis

For statistical analysis, IBM SPSS Statistics version 29 were used. Data are presented as mean ± standard deviation (SD) of mean if not otherwise indicated. Normal distribution of data was tested using Levene Test. The Mann–Whitney U test (non- parametric) or the unpaired t-test (parametric) was performed for continuously distributed variables for comparison between the two groups. Depending on the analyzed sample size, for categorical variables, either the chi-square test ($n>11$ or Fisher’s exact test ($n<10$) was performed. For assessment of more than 4 categorial variables, Pearson’s k x k test was used. A binary logistic regression analysis was used to analyze the influence of several factors on the prevalence of PCS. A p-value of  $p<$ *0.05* was considered as statistically significant.

## Results

### Demographics and clinical characteristics

**Fig. 1:**
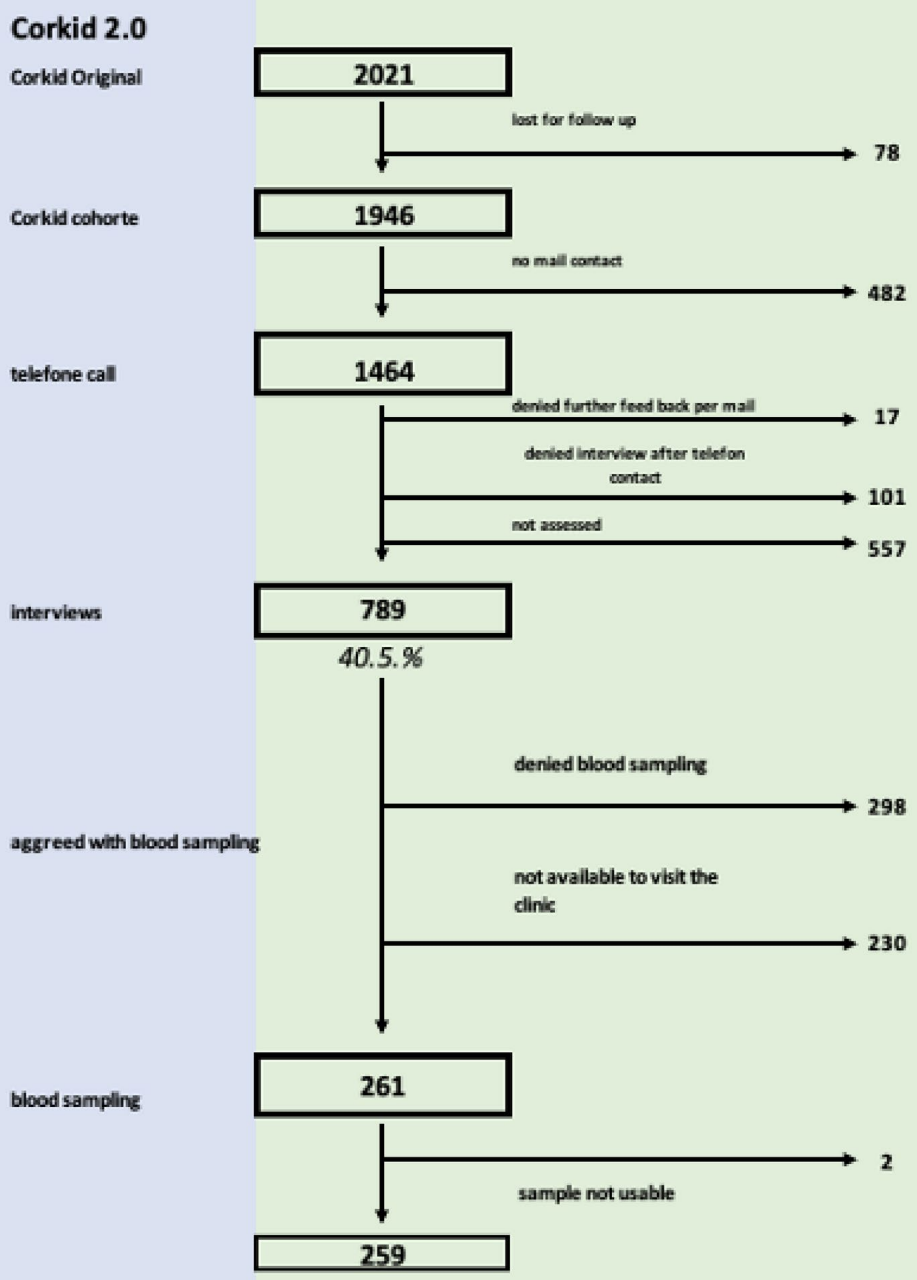
Flowchart. 2021 participants were included in the initial CorKID Study (“Corona in Kids”)^1^. Of these, 1464 were contacted by telephone and 789 were included in this study. Ab subgroup of 261 children and adolescents (33.1%) additionally received serological testing.

Of the 2121 participants included in the initial CorKID study, 1946 had consented for a follow-up interview. Of these, 1464 were contacted by telephone after a mean time of 23.6 months (range 18.5–28.9) since their first enrolment (Fig. 1). In 789 participants (358 [45.5%] females; median age 7.9 years [ICR 6.1)]) the parents were inclined to participate in a telephone interview (in 87% of cases with the mother). Demographic data of this CorKID2.0 cohort are shown in supplemental Table 1. The followed-up cohort was overall representative of the initial CorKID cohort except for the proportion of participants with an immigrant background, who were slightly underrepresented in this follow-up cohort. A detailed comparison of both cohorts is shown in supplemental Tables 2 and 3.

In addition, a subgroup of 259 participants agreed to return to the hospital for serological testing. The demographic characteristics of this subgroup were overall representative for the overall CorKID2.0 cohort, however we observed a slight overrepresentation of parents with advanced education in the group of participants coming for serological testing, although not statistically significant (*p* = *0.052*) (supp. Tab. 2).

### Timing of infections

On follow-up, 536 (67.9%) participants reported at least one, 54 (6.9%) more than one known SARS-CoV-2 infection. Of the participants with at least one reported SARS-CoV-2 infection, 19.9% were younger than five years, 31.2% between the age of five and eleven years and 16.9% twelve years or older. 253 (32.1%) participants denied any SARS-CoV-2 infection (Table 1).

**Table 1:** SARS-CoV-2 infection history and disease course.

	All	Vaccinated children	Without vaccination	*P*
N	**789 (100%)**	**368 (100%)**	**418 (100%)**	
One vaccination	44 (5.6%)	44 (12%)	–	
Two	231 (29.3%)	231 (62.8%)	–	
Three	93 (11.8%)	93 (25.3%)	–	
Children with known SARS-CoV-2 infection	536 (68%)	243 (66%)	292 (70%)	n.s
One infection	482 (61.2%)	227 (61.7%)	254 (60.9%)	
Reinfection	54 (6.9%)	16 (4.3%)	38 (9.1%)	$\textbf{P}<\textbf{0.05}$
SARS-CoV-2 infection in different age groups (missing n = 3)				
Below 6y	157 (19.9%)	30 (8.2%)	212 (50.7%)	$\textbf{P}<\textbf{0.05}$
6–11 y	246 (31.2%)	178 (48.4%)	178 (42.6%)	
12y. or older	133 (16.9%)	160 (43.5%)	28 (6.7%)	
Year of SARS-CoV-2 infection				
2020/21	106 (19.8%)	37 (35%)	69 (65%)	$\textbf{P}<\textbf{0.05}$
2022	430 (80.2%)	206 (48%)	251 (52%)	
Severity of acute SARS-CoV-2 infection				
Asymptomatic	99 (12.6%)	38 (10.3%)	61 (14.6%)	n.s
Light symptoms	383 (48.6%)	181 (49.2%)	201 (48.2%)	
Moderate	53 (6.7%)	24 (6.5%)	29 (6.9%)	
Severe/Hospitalization	2 (0.3%)	–	2 (0.5%)	
Duration of acute SARS-CoV-2 infection symptoms				
1–3 days	301 (38.1%)	125 (34%)	176 (42.1%)	n.s
4–7 days	202 (25.6%)	99 (26.9%)	102 (24.4%)	
> 7 days	26 (3.3%)	16 (4.3%)	10 (2.4%)	
Other flu-like infection	324 (41.1%)	127 (34.5%)	198 (47.4.%)	$\textbf{P}<\textbf{0.05}$

Percentages refer to the respective column.

Significant are in value [bold].

**Fig. 2:**
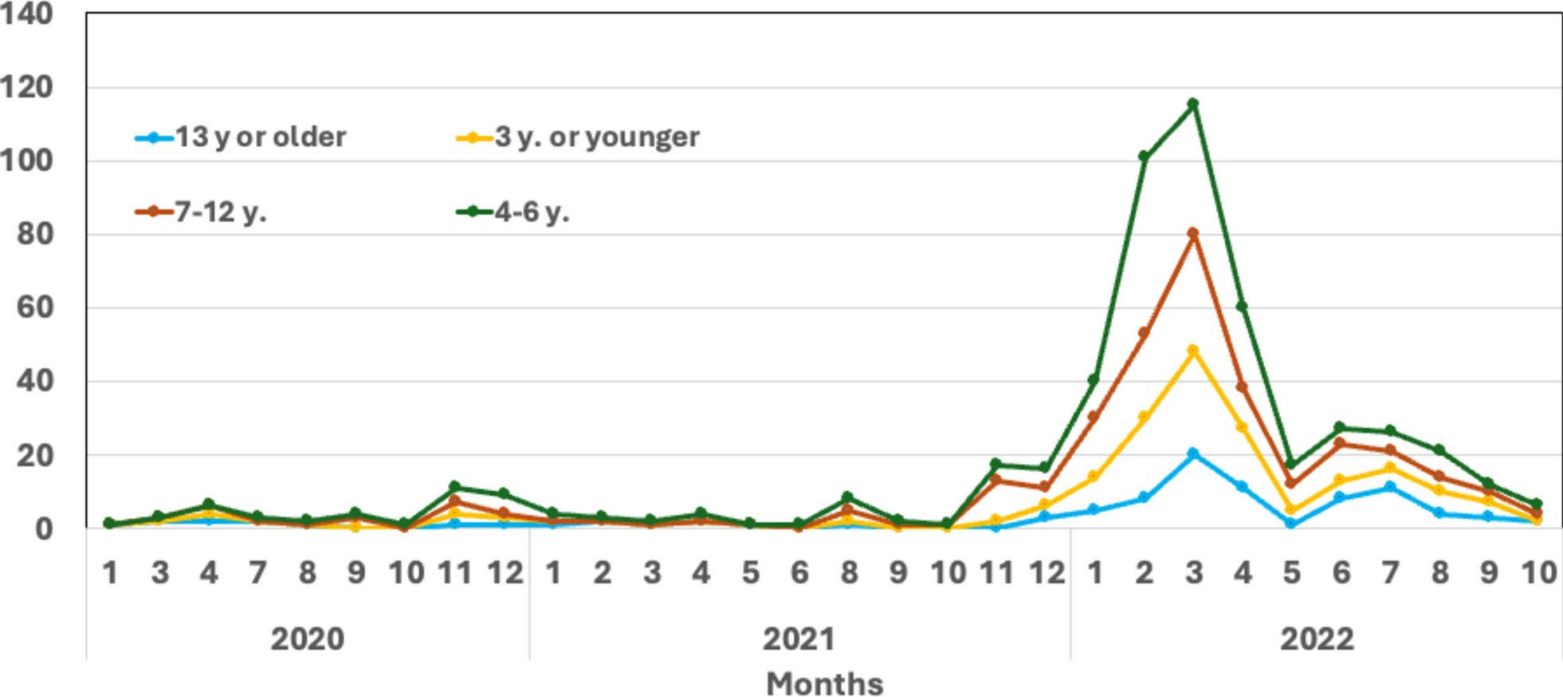
Time of SARS-CoV-2 infection. Participants were questioned regarding the timing of their first (as well as potential subsequent) SARS-CoV-2 infections. Results are shown four four different age groups as indicated.

SARS-CoV-2 infection was documented in 93% either by a positive antigen rapid test (performed in 85%) and/or PCR (66%); 7% reported typical symptoms after proved infection of their parents or siblings without SARS-CoV-2 testing on themselves. The SARS-CoV-2 infections occurred in the vast majority (n = 430; 80.2%]) in 2022 and were concentrated in the first four months of the year (Fig. 2). The predominant variant in Germany at this time was SARS-CoV-2 B.1.1.529 Omicron^7^.

### Timing of COVID-19 vaccinations

Of all 789 respondents, 368 (46.6%) reported that their children were vaccinated against SARS-CoV-2 at least once; 89% of the adolescents (≥ 12y.); 56% of children between 5 and 11 years, and 15% of children younger than 5 years, although vaccination was not recommended for this age group at that time in Germany. 75% of the non-COVID-19-vaccinated children were younger than 7 years. Of the COVID-19 vaccinated children, the vast majority received their first vaccination in the second half of 2021 (supp. Figure 1). At the time of the survey, most participants (n = 231; 62.8%) had already received two COVID-19 vaccination doses, and 93 (25.3%) had received three doses of COVID-19 vaccine. Of all COVID-19 vaccinated study participants, 52.4% experienced a SARS-CoV-2 infection following their initial COVID-19 vaccination. In the entire cohort, there was neither any difference in the frequency of SARS-CoV-2 infection, nor in the timing or duration of SARS-CoV-2 infections, between participants who were vaccinated against COVID-19 and those who were not; however, we did observe a significant lower re-infection rate in participants who were vaccinated against COVID compared with unvaccinated individuals (Table 1). In the subgroup of participants with an immigration background, we observed a significant lower SARS-CoV-2 vaccination rate as well as a statistically significant higher rate of re-infections (supp. Tab. 4).

### SARS-CoV-2 antibody assessment

In the subgroup of serologically tested individuals (n = 259), 178 (68.7%) reported having experienced a SARS-CoV-2 infection before. Infection was confirmed by the detection of SARS-CoV-2 specific nucleocapsid antibodies in 93.3% of cases. SARS-CoV-2 specific spike antibodies were detected in 98.9%. Only in two children (1.1%) SARS-CoV-2 specific antibodies were not detectable.

Consistent with the overall cohort, in the subgroup undergoing antibody assessment, one third of the parents (81 of 259 [32.1%]) denied any SARS-CoV-2 infection in their children. However, SARS-CoV-2 nucleoside antibodies were detected in 34.6% (n = 28, each 14 with or without prior vaccination) of these individuals.

With 51.4% (133 out of 259), the COVID-19 vaccination rate was slightly higher in the subgroup who came to the clinic for serological testing than in the overall CorKID 2.0 cohort. Notably, all participants (100%) exhibited a robust positive SARS-CoV-2 spike antibody titer.

**Fig. 3:**
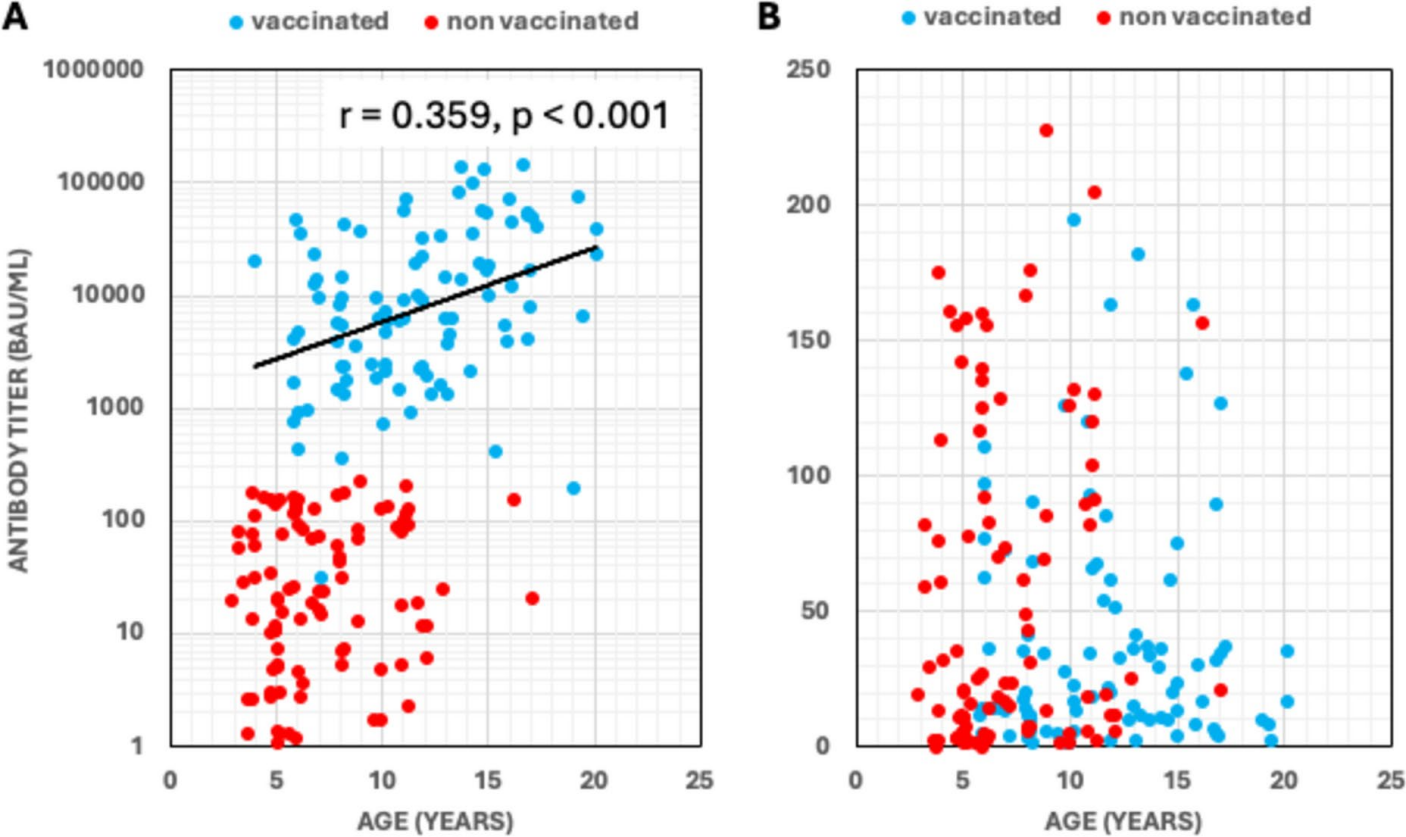
Association between SARS-CoV-2 antibody titers and age. Correlation between the serum level of anti-SARS-CoV-2 spike-antibodies (**A**) or anti-SARS-CoV-2 nucleocapsid-antibodies (**B**) and age of sampling. Data are shown for vaccinated (blue dots) and non-vaccinated (red) individuals.

For participants vaccinated against COVID-19, a positive correlation was observed between age and the titer level for spike antibodies (time interval between vaccination and blood sample was 7.9 (ICR 2.5; range: 0.5 to 21 months). This correlation was not found for participants without COVID-19 vaccination (Fig. 3A). Regarding SARS-CoV-2 nucleoside antibodies, no correlation was found between age and titer level (Fig. 3B). No statistically significant decline in antibodies was observed over time following either SARS-CoV-2 infection or COVID-19 vaccination, respectively (supp. Figure 2).

### Sequela of SARS-CoV-2 infection

SARS-CoV-2 infection was reported as mild with only minor symptoms in most participants (383 of 536 with at least one SARS-CoV-2 infection; 71.4%), and 99 (18.5%) reported that the SARS-CoV-2 infections had been completely asymptomatic. 53 participants (9.9%) had experienced moderate to more severe symptoms. In our cohort, one patient (0.19%) developed pediatric inflammatory multisystem syndrome (PIMS) with myocarditis and arterial hypotension and had long lasting sequelae. 301 (56.2%) reported that the acute illness lasted between one and three days, 202 (37.7%) had prolonged acute infection symptoms lasting between four up to seven days, and 26 (4.9%) experienced acute infection symptoms lasting for more than one week.

In 92% of cases, another family member was also infected with SARS-CoV-2 at the same time. In contrast to children, 26% of the parents reported a moderate or severe SARS-CoV-2 infection course with dyspnea or other comparable symptoms. Notably, there was a significant intra-familiar relationship: The likelihood that children’s COVID-19 symptoms were classified as severe increased if the parent experienced more a more severe COVID-19 course (OR 3.6; CI 1.4–9.3) (supp. Figure 3).

### Infections other than SARS-CoV-2

Parents were also queried regarding flu-like infections in their children within the 12 months preceding the interview. COVID-19 vaccinated children with and without SARS-CoV-2 infection had significantly fewer flu-like infections as the non-COVID-19-vaccinated group (125/243 [51.4%] versus 176/292 [61%]; OR 0.6 [CI 0.5-0.98]).

### Health status changes during the pandemic

**Fig. 4:**
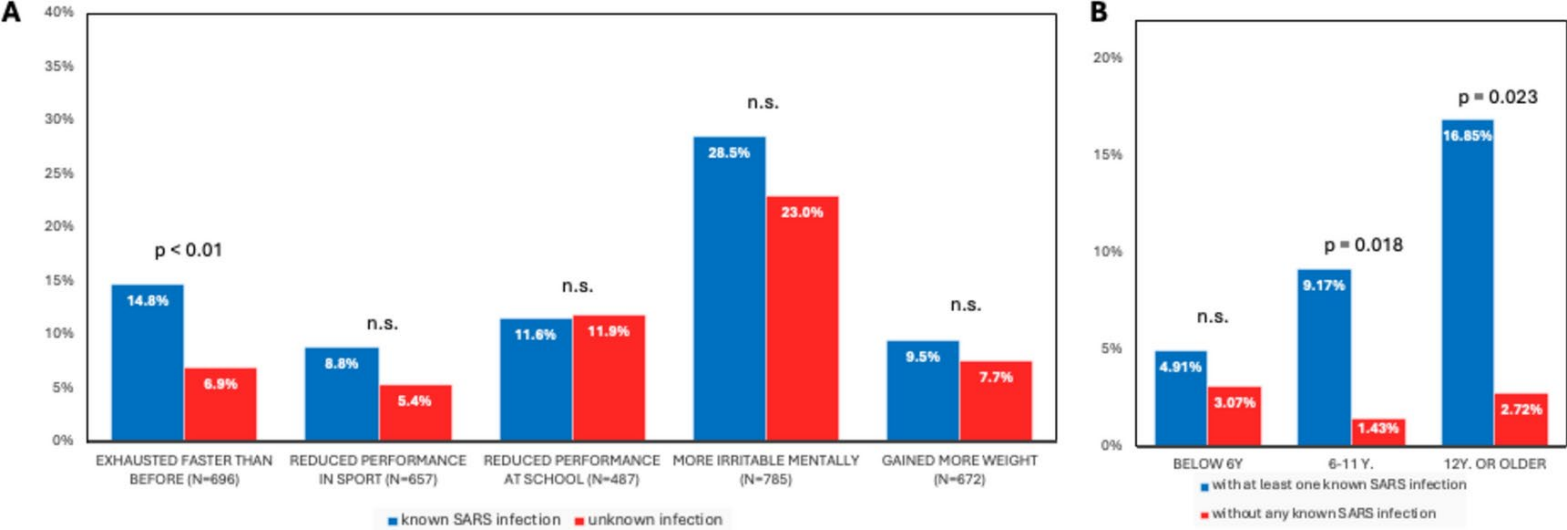
Change of children’s health state and performance compared to the time prior to the pandemic. (**A**) The parents who participated in the interviews were requested to evaluate diverse dimensions of their children’s health and functioning during the pandemic compared to pre-pandemic times. (**B**) Different age subgroups for the frequency of remarks concerning children being faster exhausted compared to their state before the pandemic. The collected responses are displayed, categorized by the infection status of the children, with instances of known SARS-CoV-2 infection (red) and instances of unknown or undetected infection (blue).

^6^Negative evaluation of current state of health, quality of life, fitness and mental state at the time of the follow-up interview was rare, only sadness was reported by one fifth of the study participants (supp. Tab. 3). Noteworthy, these answers did not differ between the study participants with or without SARS-CoV-2 infection.

Overall, no major difference regarding health status or school performance was found between children with known history of SARS-CoV-2 infection and without (Fig. 4a). However, parents of children who have experienced at least one known SARS-CoV-2 infection stated significantly more frequent that their children were exhausted faster than before the COVID-19 pandemic compared to parents of children without SARS-CoV-2 infections (14.8% vs. 6.9%, OR 2.33 [CI 1.3–4.16]). In case of children with moderate or severe symptoms (n = 55) during the acute SARS-CoV-2 infection, the percentage of fatigue increased up to 29.8% compared with 10.23% of children with known, but asymptomatic course of SARS-CoV-2-infection (n = 88; OR 3.7 [CI 1.5–9.5]). Children older than twelve years of age were mainly affected by SARS-CoV-2 infection sequela without gender differences (Fig. 4b).

### Frequency and characteristics of persistent symptoms after COVID-19 infection

**Fig. 5:**
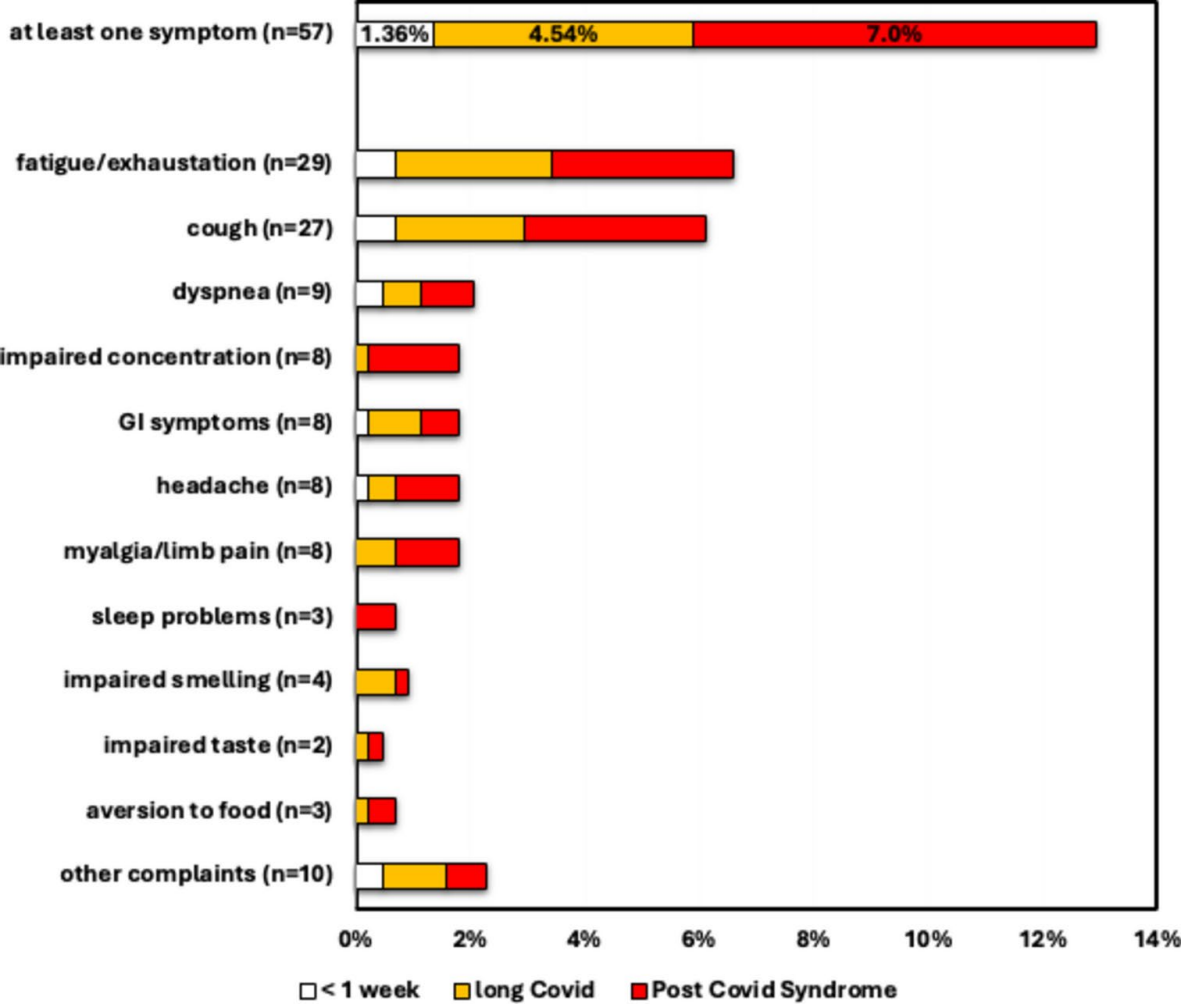
Frequency and duration of prolonged symptoms following your SARS-CoV-2 infection. For 438 children and adolescents, it was reported that they had a symptomatic COVID-19 disease course. Shown here is the frequency of mention symptoms and the duration of these symptoms that went beyond the acute symptoms (longer than 3 days). The colors indicate the duration of the symptoms: white > 3 – ≤ 7 days, yellow > 7 days – ≤ 3 months, red > 3 months.

To explore the prevalence of prolonged COVID-19 symptoms, interview responses of participants with documented SARS-CoV-2 infection dates, and interviews conducted at least three months post- SARS-CoV-2 infection were analyzed (n = 441). Among the participants who reported at least one SARS-CoV-2 infection in their children, 1.4% (n = 6) indicated that their children had experienced prolonged acute symptoms (longer than 3 days and up to 1 week), 4.8% (n = 20) reported prolonged symptoms (longer than one week and up to three months), and 7% (n = 31) reported symptoms, lasting for more than two months. The most reported prolonged symptoms after SARS-CoV-2 infection were fatigue and exhaustion followed by respiratory symptoms such as cough and dyspnea (Fig. 5).

Of 106 participants who had their first SARS-CoV-2 infection before the appearance of the SARS-CoV-2 Omicron variant, 14.2% (n = 15) retained symptoms lasting for more than three months; in contrast, only 4.8% (n = 21) of the 430 people with an SARS-CoV-2 infection during the Omicron era (*p* $<$ *0.01*). Since no COVID-19 vaccine was available for children in the first year of the pandemic, only 35% of children were vaccinated against COVID-19 compared to 47% in the later period. Logistic regression analysis with COVID-19 vaccination status and SARS-CoV-2 virus variant as independent variables revealed that only the virus viriant significantly influenced PCS (*p* $<$*0.01*).

**Fig. 6:**
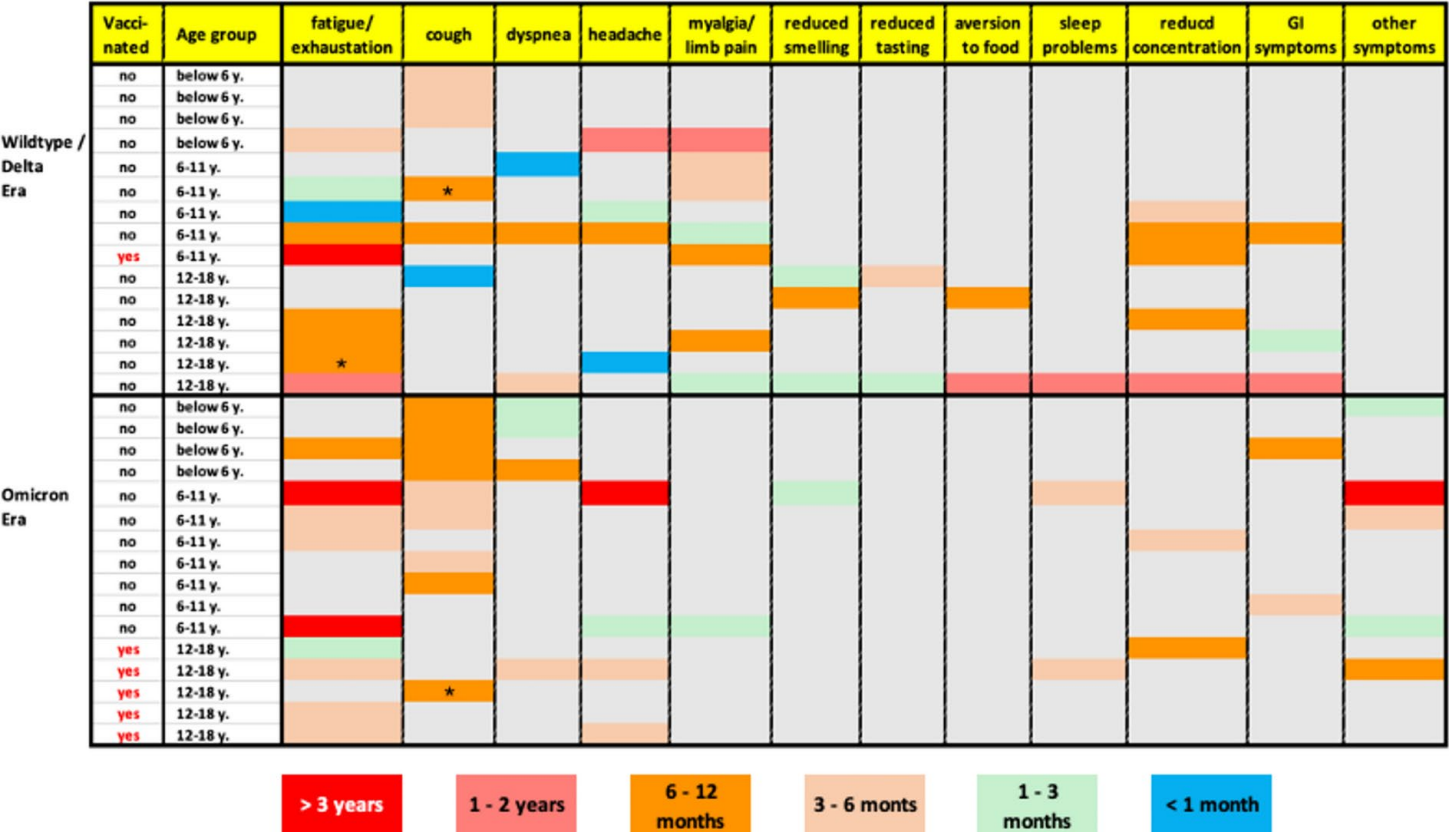
Post COVID syndrome (PCS)-like symptoms. A total of 31 participants reported that symptoms persisted for more than 3 months after SARS-CoV-2 infection. Shown here are the symptoms of the individual participants and the duration of each symptom. The cohort is divided by age group and time of infection (SARS-CoV-2 wild-type or Delta variant [infection before December 2021] or during the Omicron era). * indicates that the symptoms were still present at the time of the follow-up interview in 2022 and that these patients could not be reached at the second follow-up interview in 2024.

Figure 6 illustrates the diverse symptom patterns for PCS-like symptoms, varying in symptom type and duration, influenced by the timing of SARS-CoV-2 infection and the age of the study participant. It is noteworthy that certain symptoms may endure briefly in many children, whereas others may persist for several years in the same individual. For instance, fatigue or rapid exhaustion, resolved within a year in 11 children but persisted longer in 7 others. Respiratory symptoms typically disappeared within a few months for most cases but resolved in all cases within a year. Furthermore, it seems interesting to note that impaired sense of taste and smell occurred almost exclusively in infections that predated the onset of the Omicron variant.

In this cohort, three children continued to experience PCS-like symptoms after COVID-19 within the Omicron period for nearly four years by 2024. The beforementioned non-vaccinated male child who developed Paediatric Inflammatory Multisystem Syndrome (PIMS) in March 2022, still grapples with heart issues, persistent fatigue, and recurring migraine attacks after a mild SARS-CoV-2 infection. A second female child (11 y.), who has a pre-existing autistic disorder, saw a worsening of autistic symptoms. Despite vaccination, the girl later experienced two new SARS-CoV-2 infections, with moderate symptoms. Furthermore, a third non-vaccinated child (8 y) experienced symptoms such as fatigue and limb pain after a mild SARS infection that had persisted for over two years compatible with ME/CFS.

## Discussion

This study reports the findings of a long-term follow-up, investigating SARS-CoV-2 seroconversion rates and SARS-CoV-2 infection sequela in a population-based cohort study during the pandemic in everyday life conditions. Key results include: (1) 68% of participants reported at least one SARS-CoV-2 infection, mostly in 2022, characterized by predominantly mild illness; (2) the vast majority of serologically tested participants exhibited detectable SARS-CoV-2 spike and nucleoside antibodies; (3) undetected SARS-CoV-2 infections occurred in approximately 30% of participants across all age groups; (4) 47% of participants received at least one COVID-19 vaccination, which was associated with fewer flu-like infections; (5) prolonged symptoms were reported in 7% and few participants had persisiting symptoms for up to four years including fatigue and reduced physical performance.

Between June 2020 and February 2021, we conducted a population-based cohort study involving asymptomatic children and their parents to determine the SARS-CoV-2 seroconversion rate during this period. At at that time, only 58 children tested positive for SARS-CoV-2^1^. Subsequently, with the advent of the Omicron variant in early 2022, infection rates surged across the population. Therefore, we conducted a follow-up of this cohort approximately 2 years later to investigate the evolution of seroconversion rates within this population based cohort.

With a seroconversion rate against SARS-CoV-2 of over 90%, our results are in line with findings from other countries and another data from Germany^8–10^. While in the time period before omicron predominance seroprevalence rates in children in European countries differed due to the extent of contact restrictions and generally showed higher seroconversion rates in older children and adolescents^11,12^, seroconversion rates in European countries rose similarly in the Omicron era^13,14^. Noteworthy, within our pediatric population, SARS-CoV-2 seroprevalence rates showed marked differences among age groups: Among school-aged children and adolescents, SARS-CoV-2 antibodies were detectable in 97% in the adolescents and among children under the age of six in around 90% of cases. An additional remarkable result of our study was that antibodies against the SARS-CoV-2 nucleoside antibodies were found in about one third of the children who reported not having experienced any SARS-CoV-2 infection. In line with this high level of unnoticed SARS-CoV-2 infections, the vast majority who had history of SARS-CoV-2 infection reported mild with only moderate symptoms. About 20% of the COVID-19 group had a positive SARS-CoV-2 test but an asymptomatic disease course and it should be emphasized that none of the study participants required hospitalization for acute SARS-CoV-2 infection.These data suggest that the acute course of SARS-CoV-2 infection during omicron in children was rather mild, when compared with previous SARS-CoV-2 variants^15^; an observation that is further supported by previous findings by Engels et al.^16^.

Concurrently to an increase in SARS-CoV-2 infections, COVID-19 vaccination became available for children and adolescents around the same time and contributed to an increase in seroprevalence. In Germany, COVID-19 vaccinations have been recommended for children and adolescents of 12 years and older in August 2021 and in May 2022 for healthy children 5–11 years of age^17^. At the time of our study, the baseline immunization rate against COVID-19 in Germany was 19.9% for children 5–11 years of age and 69.4% for adolescents 12–17 years of age^18^. In our cohort, the vaccination rate for adolescents was 89% and 56% for children. For children younger than 5 years, Comirnaty and Spikevax had been recently approved by the European Medicines Agency (EMA)^17^; however there was no recommendation of the Standing Committee on Vaccination (STIKO) in Germany at the time of the study. It is thus noteworthy that in our cohort, 15% of children younger than 5 years have nevertheless already received an off-label vaccination against SARS-CoV-2. Upon serological testing, we detected spike antibodies in 100% of participants who claimed to have received a COVID-19 vaccination and underwent serological testing.

In concordance with observations in other European countries, we found that children and adolescents with immigrant background are both at higher risk for re-infection and and less likely to be vaccinated compared with the general population^1,19^. Reasons might include impaired access to healthcare facilities as well as concerns regarding the safety of the vaccine^20,21^.

In addition to the acute symptoms of SARS-CoV-2 infection, Long- and Post-COVID symptoms have increasingly become the focus of both scientific and public debate in recent times^22^. Symptoms, lasting more than 3 months after acute SARS-CoV-2 infection, were reported in 7% of study participants, in line with other population based data e.g. from Denmark^23,24^. The most frequent symptoms were reduced physical perfomance and fatigue, in line with the findings in other cohorts^22,24,25^, whereas hyposmia did not play a role in this cohort of children after infection with the SARS-CoV-2 omicron strain. Long and post COVID symptoms have been reported previosuly in children of all ages. The most common described symptoms included fatigue, headache, dizziness, dyspnoea, chest pain, dysosmia, dysgeusia, reduced appetite, concentration difficulties, memory issues, mental exhaustion, physical exhaustion and sleep issues^22^ . Data on the frequency and duration of these symptoms varied in the literature as conclusive assessment is complicated by different methods and disease definitions.

Although we did not observe a significant difference in SARS-CoV-2 infection rates between COVID-19 vaccinated and non-COVID-19-vaccinated children and adolescents, COVID-19 vaccination was associated with a reduced re-infection rate and with a decreased prevalence of prolonged symptoms by trend. Furthermore, COVID-19 vaccinated children with or without evidence of SARS-CoV-2 infection reported approximately 12% less flu like infections during the observation period. Unspecific immunmodulatory effects preventing other infections have been described after other vaccinations e.g. with BCG^26,27^. It is thus tempting to speculate, that similar effects also play a role in preventing other viral infections in COVID-19 vaccinated children and adolescents. However, to our knowledge this is the first observation in response to mRNA vaccines and needs to be further evaluated.

Impaired general health and well-being as well as increased anxiety and depression disorders in children and adolescents during the COVID-19 pandemic had been reported before^28^. In addition, reduced physical perfomance, fatigue, headache and reduced ability to concentrate had been decribed in both adolectens with and without history of SARS-CoV-2 infection^29^. When comparing children with and without confirmed SARS-CoV-2 infection in our cohort, significant differences were only found for fatigue and weight gain which were more frequent in individuals with a histroy of COVID-19. Thus, these data suggest that some symptoms reported in the course of the pandemic cannot be attributed exclusively to SARS-CoV-2 infections, but that other measures such as school closures and quarantine regulations during COVID-19 pandemic must also be taken into account.

## Limitations

Limitations of this study include the single-center design and the smaller sample size, compared with the initial CorKID study. In addition, there could be a potential selection-bias in favor of rather cautious and concerned parents who were willing to participate in this study; in fact, children of well educated parents were slightly overrepresented in this follow-up study, when compared with the initial CorKID study. However, we found that the follow-up cohort was widely representative for the initial cohort in terms of children-related features. Another confine to consider is that, prolonged symptoms were not diagnosed in a standardized way, but based on structured interviews, which were conducted with the children’s parents. Furthermore, serological data on SARS-Cov-2 infection and COVID-19 vaccination were only validated in a subgroup of pediatric participants who volunteer for another blood examination. However, this subgroup was representative for the entire cohort, thus we consider it acceptable to apply these results to the entire study cohort. In addition, a PCR test was not strictly required for the diagnosis of SARS-CoV-2 infection but positive antigen detection tests and a positive SARS-CoV-2 test of another family member and parallel symptomatic children were also included. Of note, this approach is in line with the official regulations at the time for dealing with infections or suspected cases during the pandemic in Germany, and thus reflects the real world condition in the study.

## Conclusion

During two years of the pandemic, the majority of children and adolscents aquired an immune response to SARS-CoV-2 by COVID-19 vaccination and/or SARS-CoV-2 infection. Most SARS-CoV-2 infections occured in 2022 during the predominance of the omicron variant prevalent in Germany. Most SARS-CoV-2 infections were asymptomatic or mild, but about 7% of the participants experienced prolonged symptoms post infection, mainly fatigue and exhaustian. COVID-19 vaccinations seemed to reduce the risk of other infections during the follow up.

## Supplementary Information

Supplementary Information.

## Data Availability

The datasets used and/or analysed during the current study available from the corresponding author on reasonable request.

## Trial registration

German Registry for Clinical Trials Identifier Bochum: DRKS00022434 (registration:07.08.2020), Dresden: DRKS 00022455 (registration: 23.07.2020).
