# Supplementary material for: Long-term effects of SARS-CoV-2 infection and vaccination in a population-based pediatric cohort
Source: Sci Rep. 2025 Jan 23;15:2921. doi: 10.1038/s41598-024-84140-6 (PMC11758015; doi:10.1038/s41598-024-84140-6)
Supplement: Supplementary file 1 — Supplementary Information. [file 41598_2024_84140_MOESM1_ESM.docx]

Supp. Material

CorKID 2.0: Long-term effects of SARS-CoV-2 infection and vaccination in a population-based pediatric cohort


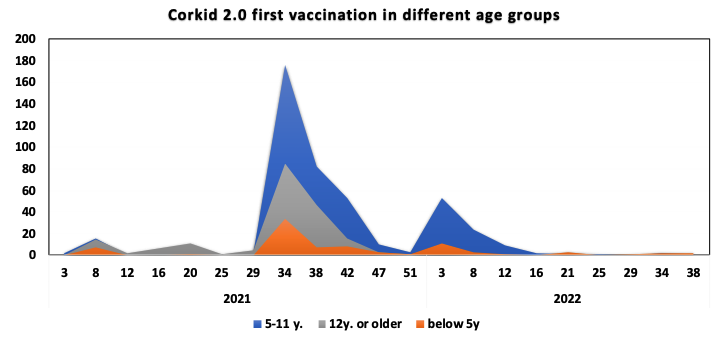


**Supp. Fig. 1: Time of 1^st^ vaccination in four different age groups.** Shown on the x-axis are the number of cases, on the y-axis the calendar week of the respective year. Data are shown for age groups < 5 years, 5-11 years and > 12 years, as indicated in the figure.

**
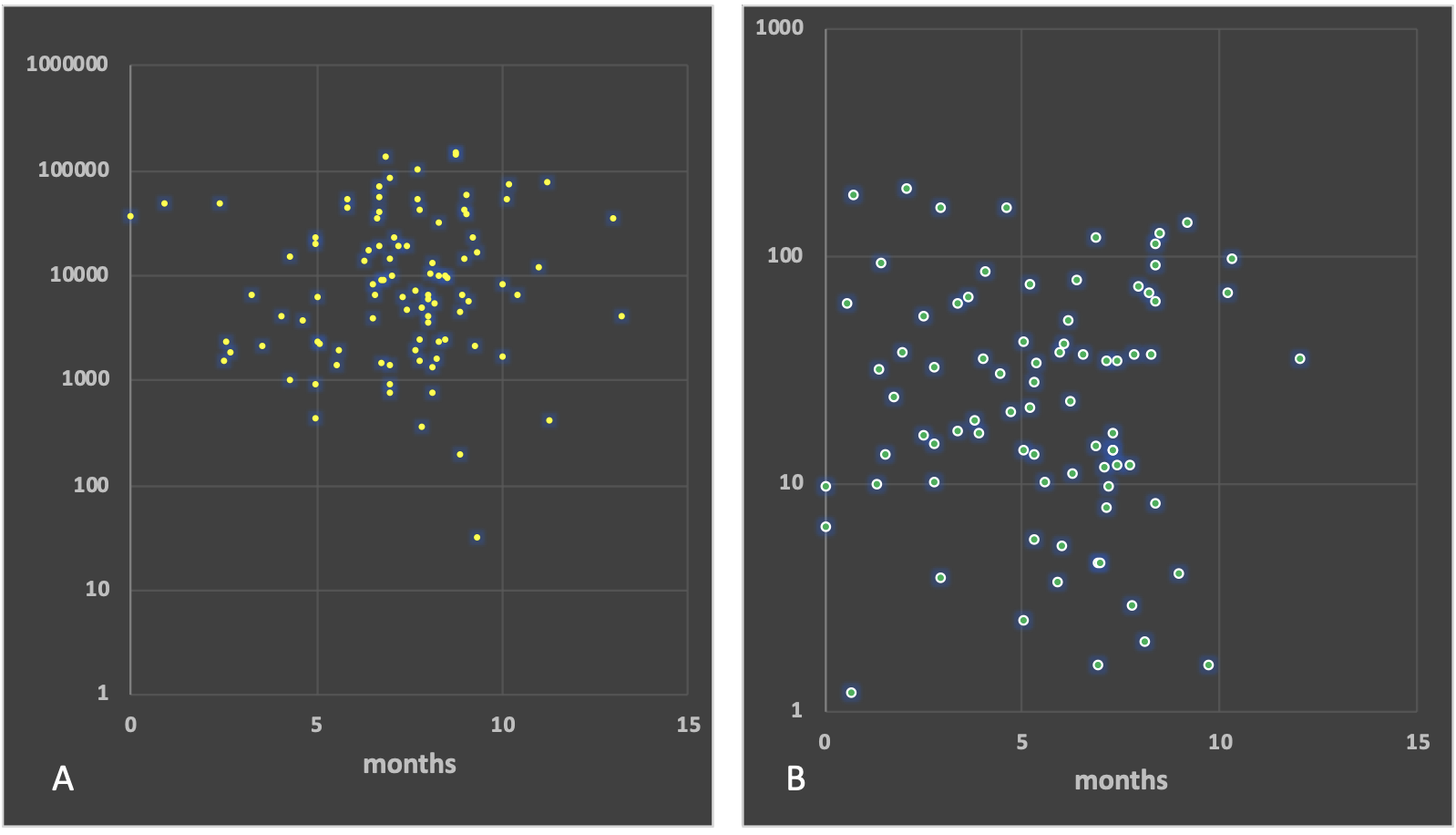
**

**Antibody Titer**

**Supp. Fig. 2: Relationship between anti-SARS-CoV-2 antibody serum titer and time interval to vaccination.** Shown are the antibody serum titers for anti-SARS-CoV-2 spike antibodies (A) and anti-SARS-CoV-2 nucleocapsid antibodies (B) plotted against the time interval to vaccination (months).

**
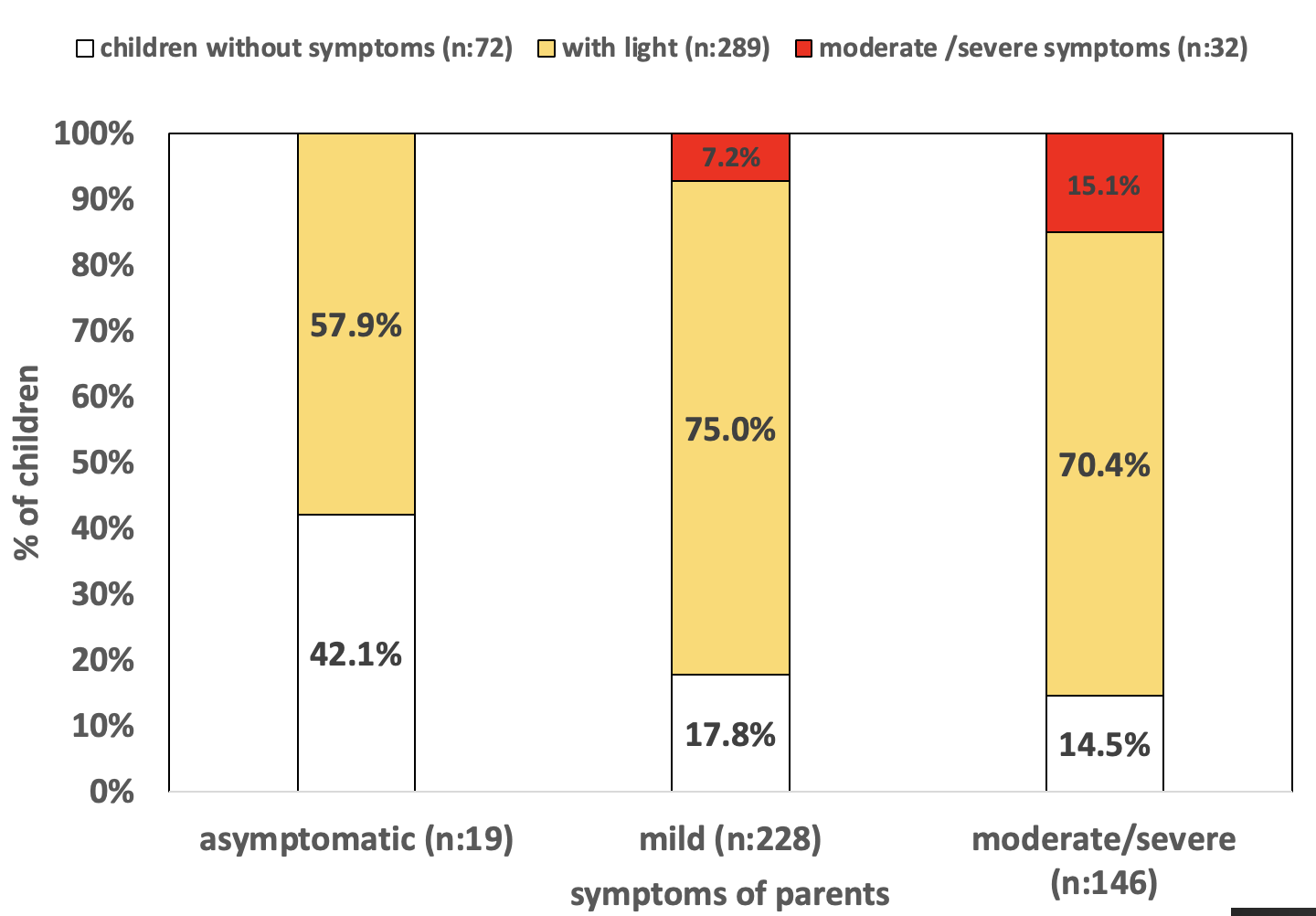
**

**Supp. Fig. 3: Intra-familial symptom burden.** In addition to asking about their children's symptoms, interviewed parents were queried about their own potential SARS-CoV-2 infections and the severity of their illness (asymptomatic, mild, or moderate/severe) during the interview. This figure illustrated the occurrence rates of asymptomatic (white), mild (yellow), or moderate / severe (red) SARS-CoV-2 infections in all children (x-axis), grouped by the severity of the SARS-COV-2 infection of their parents (y-axis).

| **Supp. Table 1: Demographic characteristics of the CorKID follow-up cohort** | |
| --- | --- |
| children (N) | 789 (100%) |
| out of families (N) | 753 |
| Follow-up (months; mean, range) | 23.6 (18.5, 28.9) |
| female | 358 (45.5%) |
| Age at time of follow-up (median;IQR) | 7.9 (6.1) |
| below 6y | 243 (30.8%) |
| 6-11 y. | 357 (45.2%) |
| 12y. or older | 189 (24%) |

| **Supp. Table 2: Comparison of demographic characteristics between initial CorKID Cohort, CorKID Follow-up Cohort and Subgroup with serology** | | | |  |  |
| --- | --- | --- | --- | --- | --- |
|  | **initial CorKID cohort** | **Corikd 2.0** | |  |  |
|  |  | **Interview** | **plus serology** |  |  |
| **Children (N)** | 1945 (100%) | 789 (100%) | 259 (100%) | |  |
| out of families (N) | 1906 | 753.0 | 235 | |  |
| **Follow-up** (months; mean, range) | - | 23.6 (18.5...28.9) | 23.6 (18.5...28.9) | |  |
| median (IQR) |  | 23.8 (3.3) | 23.6 (3.2) | |  |
| **Sex** |  |  |  | |  |
| female | 912 (46.9%) | 358 (45.4%) | 126 (48.6%) | |  |
| Age | | | | | |
| At time of initial CorKID inclusion | 7.1(±4.7) |  |  | |  |
| 3 y. or younger | 479 (24.6%) | - | - | |  |
| 4-6 y. | 679 (34.9%) | - | - | |  |
| 7-12 y. | 379 (19.5%) | - | - | |  |
| 13 y or older | 391 (20.1%) | - | - | |  |
| **Age at time of follow-up (median;IQR)** |  | 7.9 (6.1) | 8 (6) | |  |
| below 6y |  | 243 (30.8%) | 78 (30.1%) | |  |
| 6-11 y. |  | 357 (45.2%) | 120 (46.3%) | |  |
| 12y. or older |  | 189 (24%) | 61 (23.6%) | |  |
| Preexisting diseases (before initital CorKID inclusion) | | | | | |
| Any | 169 (8.7%) | 68 (8.6%) | 23 (8.9%) | |  |
| Severe | 3 (0.2%) | 0 (0%) | 0 (0%) | |  |
| Moderate | 126 (6.5%) | 53 (6.7%) | 18 (6.9%) | |  |
| Low | 40 (2.1%) | 15 (1.9%) | 5 (1.9%) | |  |
| Current steroid or immmunsupressive treatment | 94 (4.8%) | 43 (5.4%) | 15 (5.8%) | |  |
| Highest school degree of parents | | | | | |
| Advanced technical college entrance qualification / Abitur | 1285 (67.4%) | 599 (79.5%) | 213 (90.6%) | |  |
| Intermediate school diploma | 385 (20.2%) | 125 (16.6%) | 29 (12.3%) | |  |
| Secondary school diploma | 116 (6.1%) | 38 (5%) | 10 (4.3%) | |  |
| No degree | 45 (2.4%) | 11 (1.5%) | 4 (1.7%) | |  |
| **Immigration background** | 692 (36.3%) | 242 (32.1%) | 61 (26%) | |  |
| Turkey |  |  |  | |  |
| Middle East | 160 (8.4%) | 40 (5.3%) | 6 (2.6%) | |  |
| Asia | 85 (4.5%) | 20 (2.7%) | 3 (1.3%) | |  |
| NIS | 26 (1.4%) | 14 (1.9%) | 3 (1.3%) | |  |
| Eastern Europe | 104 (5.5%) | 27 (3.6%) | 5 (2.1%) | |  |
| Southern Europe | 241 (12.6%) | 94 (12.5%) | 25 (10.6%) | |  |
| other | 44 (2.3%) | 27 (3.6%) | 14 (6%) | |  |
| **Persons/Household** |  |  |  | |  |
| 1-3 | 486 (25.5%) | 196 (26%) | 61 (26%) | |  |
| 4-5 | 1202 (63.1%) | 534 (70.9%) | 180 (76.6%) | |  |
| more than 5 | 137 (7.2%) | 42 (5.6%) | 14 (6%) | |  |

| **Supp. Table 3: Comparison of demographic characteristics of participants with and without immigration background within the CorKID2.0 Follow-up Cohort** | | | | |
| --- | --- | --- | --- | --- |
|  | **Immigrant background** | | **OR (CI 95I)** | **p (overall)** |
|  | yes | **no** |  |  |
| **N** | 242 | 532 |  |  |
| **Age** | | | | |
| below 6y | 53 (21,9%) | 131 (24,6%) | 0,86 (0,6…1,23) | n.s. |
| 6-11 y. | 85 (35,1%) | 188 (35,3%) | 0,99 (0,72…1,36) |  |
| 12y. or older | 61 (25,2%) | 115 (21,6%) | 1,22 (0,86…1,74) |  |
| **female** | 114 (47,1%) | 237 (44,5%) | 1,11 (0,82…1,5) |  |
| **Preexisting diseases** (before initital CorKID inclusion) | | | | |
| Any | 16 (6,6%) | 51 (9,6%) | 0,67 (0,37…1,2) | n.s. |
| severe | - | - | - |  |
| moderate | 12 (5%) | 40 (7,5%) | 0,64 (0,33…1,25) |  |
| mild | 4 (1,7%) | 11 (2,1%) | 0,8 (0,25…2,53) |  |
| **Highest school degree of parents** | | | | |
| Advanced technical college entrance qualification / Abitur | 175 (72,3%) | 424 (79,7%) | 0,67 (0,47…0,95) | n.s |
| Intermediate school diploma | 44 (18,2%) | 81 (15,2%) | 1,24 (0,83…1,85) |  |
| Secondary school diploma | 18 (7,4%) | 20 (3,8%) | **2,06 (1,07…3,96)** |  |
| No degree | 5 (2,1%) | 6 (1,1%) | 1,85 (0,56…6,12) |  |
| **Persons/Household** | | | | |
| 1-3 | 85 (35,1%) | 105 (19,7%) | **2,2 (1,57…3,09)** | n.s. |
| 4-5 | 111 (45,9%) | 274 (51,5%) | 0,8 (0,59…1,08) |  |
| more than 6 | 46 (19%) | 153 (28,8%) | 0,58 (0,4…0,84) |  |
| **Number of rooms of the apartment** | | | | |
| 1-3 | 52 (21,5%) | 144 (27,1%) | 0,74 (0,51…1,06) | n.s. |
| 4-5 | 171 (70,7%) | 363 (68,2%) | 1,12 (0,8…1,56) |  |
| more than 5 | 18 (7,4%) | 24 (4,5%) | 1,7 (0,9…3,2) |  |
| **Rooms / Person in household** | | | | |
| 1 or more rooms/person | 155 (64%) | 457 (85,9%) | **0,29 (0,2…0,42)** | p< 0.001 |
| less 1 room/person | 86 (35,5%) | 74 (13,9%) | **3,41 (2,38…4,89)** |  |

| **Supp. Table 4: Comparison of SARS-CoV-2 infection history and disease course between participants with and without immigration background within the CorKID2.0 Follow-up cohort** | | | | |
| --- | --- | --- | --- | --- |
|  | **Immigrant background** | | **OR (CI 95I)** | **p (overall)** |
|  | **yes** | **no** |  |  |
| **N** | 242 | 532 |  |  |
| **Vaccination** | 79 (32,6%) | 284 (53,4%) | **0,42 (0,31…0,58)** | p< 0.05 |
| One vaccination | 12 (5%) | 32 (6%) | 0,82 (0,41…1,61) |  |
| Two | 56 (23,1%) | 172 (32,3%) | **0,63 (0,44…0,89)** |  |
| Three | 11 (4,5%) | 80 (15%) | **0,27 (0,14…0,52)** |  |
| **Reported SARS- CoV-2 infection** | 170 (70,2%) | 355 (66,7%) | 1,18 (0,85…1,64) | p< 0.05 |
| only one infection | 145 (59,9%) | 326 (61,3%) | 0,94 (0,69…1,29) |  |
| re-infection | 25 (10,3%) | 29 (5,5%) | **2 (1,14…3,49)** |  |
| 1. SARS infection before 1. vaccination | 18 (7,4%) | 32 (6%) | 1,26 (0,69…2,28) |  |
| 1. SARS infection before last vaccination | 42 (17,4%) | 136 (25,6%) | **0,61 (0,42…0,9)** |  |
| last SARS infection at least 14 days after vaccination | 40 (16,5%) | 132 (24,8%) | **0,6 (0,41…0,89)** |  |
| **Complains of acute SARS-CoV-2 infection** | | | | |
| asymptomatic | 31 (12,8%) | 66 (12,4%) | 1,04 (0,66…1,64) | n.s. |
| light symptoms | 121 (50%) | 253 (47,6%) | 1,1 (0,81…1,49) |  |
| moderate/severe (*) | 18 (7,4%) | 35 (6,6%) | 1,14 (0,63…2,06) |  |
| **Duration of complains** | | | | |
| 1-3 days | 97 (40,1%) | 199 (37,4%) | 1,12 (0,82…1,53) | n.s. |
| 4-7 day | 61 (25,2%) | 135 (25,4%) | 0,99 (0,7…1,41) |  |
| Up to 3 months | 9 (3,7%) | 17 (3,2%) | 1,17 (0,51…2,66) |  |
| **Other infection than SARS-CoV-2** | 102 (42,1%) | 219 (41,2%) | 1,04 (0,77…1,42) | n.s. |
| after SARS | 64 (26,4%) | 135 (25,4%) | 1,06 (0,75…1,49) |  |
| before SARS | 3 (1,2%) | 3 (0,6%) | 2,21 (0,44…11,05) |  |
| other, but no SARS | 35 (14,5%) | 81 (15,2%) | 0,94 (0,61…1,45) |  |
| **SARS infections within the family / household** | 84 (34,7%) | 182 (34,2%) | 1,02 (0,74…1,41) | n.s. |

| **Supp. Table 5: Change in health status depending on infection history** | | | |
| --- | --- | --- | --- |
|  | **known SARS infection** | **unknown infection** | **OR (CI)** |
| N | 536 (100%) | 253 (100%) |  |
| **changes of child's condition compared to the time before the pandemic**  (n=number of answers;% of agreeing answers)) | | | |
| exhausted faster than before (n=696) | 71 (14.8%) | 15 (6.9%) | 2.33 (1.3…4.16) |
| reduced performance in sport (n=657) | 40 (8.3%) | 11 (5.1%) | 1.69 (0.85…3.36) |
| reduced performance at school (n=487) | 40 (8.3%) | 17 (7.9%) | 0.98 (0.53…1.78) |
| more irritable mentally (n=785) | 128 (26.7%) | 47 (21.8%) | 1.33 (0.91…1.96) |
| gained more weight (n=672) | 45 (9.4%) | 17 (7.9%) | 1.27 (0.71…2.27) |
| **784** |  |  |  |
| **global health** (n=783) |  |  |  |
| excellent | 142 (26.7%) | 72 (28.6%) |  |
| very good | 209 (39.3%) | 108 (42.9%) |  |
| good | 144 (27.1%) | 57 (22.6%) |  |
| less good | 33 (6.2%) | 13 (5.2%) |  |
| bad | 4 (0.8%) | 2 (0.8%) |  |
| very bad | 0 (0%) | 0 (0%) |  |
| less good to very bad | 37 (7%) | 15 (6%) | 1,18 (0,64…2,19) |
| **quality of life** (n=784) |  |  |  |
| excellent | 136 (25.6%) | 62 (24.6%) |  |
| very good | 223 (41.9%) | 94 (37.3%) |  |
| good | 159 (29.9%) | 87 (34.5%) |  |
| less good | 14 (2.6%) | 7 (2.8%) |  |
| bad | 1 (0.2%) | 2 (0.8%) |  |
| very bad | 0 (0%) | 0 (0%) |  |
| less good to very bad | 15 (2.8%) | 9 (3.6%) | 0.78 (0.34…1.8) |
| **mental state** (n=777) |  |  |  |
| excellent | 96 (18%) | 36 (14.3%) |  |
| very good | 168 (31.6%) | 87 (34.5%) |  |
| good | 218 (41%) | 102 (40.5%) |  |
| less good | 46 (8.6%) | 21 (8.3%) |  |
| bad | 2 (0.4%) | 2 (0.8%) |  |
| very bad | 0 (0%) | 0 (0%) |  |
| less good to very bad | 48 (9%) | 23 (9.1%) | 0.97 (0.58…1.63) |
| **fitness** (n=782) |  |  |  |
| very good | 242 (45.5%) | 111 (44%) |  |
| good | 176 (33.1%) | 88 (34.9%) |  |
| at most moderate | 68 (12.8%) | 34 (13.5%) |  |
| a little | 28 (5.3%) | 10 (4%) |  |
| no | 18 (3.4%) | 8 (3.2%) |  |
| at most moderate to no fitness | 46 (8.6%) | 18 (7.1%) | 1.22 (0.69…2.15) |
| **Feeling full of energy** (n= 784 ) |  |  |  |
| always | 234 (44%) | 115 (45.8%) |  |
| often | 199 (37.4%) | 79 (31.5%) |  |
| sometimes | 69 (13%) | 35 (13.9%) |  |
| rare | 23 (4.3%) | 19 (7.6%) |  |
| never | 8 (1.5%) | 2 (0.8%) |  |
| less good to very bad | 31 (5.8%) | 21 (8.4%) | 0.67 (0.38…1.2) |
| **Sadness** (n = 767) |  |  |  |
|  |  |  |  |
| never | 299 (56.2%) | 130 (51.8%) |  |
| rare | 108 (20.3%) | 59 (23.5%) |  |
| sometimes | 97 (18.2%) | 42 (16.7%) |  |
| often | 15 (2.8%) | 16 (6.4%) |  |
| always | 1 (0.2%) | 0 (0%) |  |
| sometimes to always | 113 (21.2%) | 58 (23.1%) | 0.9 (0.63…1.3) |
| **Loneliness** (n = 748) |  |  |  |
| never | 400 (75.2%) | 174 (69.3%) |  |
| rare | 64 (12%) | 41 (16.3%) |  |
| sometimes | 29 (5.5%) | 20 (8%) |  |
| often | 12 (2.3%) | 8 (3.2%) |  |
| always | 0 (0%) | 0 (0%) |  |
| sometimes to always | 41 (7.7%) | 28 (11.2%) | 0.68 (0.41…1.13) |
